# P-2035. Comparison between Community and Hospital Onset Clostridioides difficile Infection in a Safety Net Hospital

**DOI:** 10.1093/ofid/ofaf695.2199

**Published:** 2026-01-11

**Authors:** Lujain Malkawi, Eryiel Mascardo, Susanna Paschal, An-Lin Cheng, Ume Abbas

**Affiliations:** Department of Medicine, Division of Infectious Diseases, University of Missouri-Kansas City, Kansas City, Missouri, Kansas City, MO; University of Missouri - Kansas City, Kansas City, Missouri; Department of Infection Control and Prevention, University Health, Kansas City, Missouri., Kansas City, Missouri; University of Missouri-Kansas city, Kansas city, Missouri; University of Missouri-Kansas City, Kansas City, Missouri, Kansas City, Missouri

## Abstract

**Background:**

University Health (UH) Medical Center is a longstanding 238-bed safety net hospital in Kansas City, MO, that uses LabID Event Reporting for Clostridioides difficile infection (CDI), with pre-agreed intuitional testing criteria. We compared the characteristics of hospital-onset (HO) versus community-onset (CO) cases.

**Methods:**

We conducted a retrospective cohort study that included all patients with a positive polymerase chain reaction (PCR) for toxigenic C. difficile in 2024. We defined CDI occurring on hospital day > 3 as HO and ≤ 3 as CO. Data included demographic and epidemiological variables, comorbidities, onset of diarrhea and timing of stool collection, length of stay (LOS) and exposures (within past six months of CDI) to hospitalization, surgery, and/or medications including laxatives, proton-pump inhibitors, immunosuppressants and antimicrobials. Continuous variables were compared via Mann-Whitney U or t test. Categorical variables were compared using X2 or Fisher’s exact test. Statistical significance was at p< 0.05.

**Results:**

In 2024 there were 120 CDIs, of which 100 were CO and 20 were HO. Cases with HO-CDI had higher LOS (18 vs 4 days, p< 0.001) and ATLAS scores (4 vs 2, p=0.002), while CO-CDI cases had higher mean albumin (3.5 vs 2.9 g/dL, p=0.008). HO-CDI compared to CO-CDI had a higher proportion of cases with recent procedures or surgeries (55% vs 15%, p< 0.001), presence of infection (90% vs 42%, p< 0.001), fever (65% vs 18%, p< 0.001), and use of overall (95% vs 57%, p=0.001) and multiple antibiotics (85% vs 26%, p< 0.001). Tube-feeding (25% vs 1%) and laxative-use (65% vs 7%) were also higher for HO-CDI. (p< 0.001). Conversely, bowel disease was more prevalent among CO-CDI cases (24% vs 0%, p=0.012). Other factors such as gender, prior history of CDI, immunosuppression, specialty consultations and CDI treatment (100% vs 93%, p=0.599) were not significantly different between the groups.

**Conclusion:**

The contribution of CO-CDI cases to CDI episodes and treatment was substantial at UH. Cases with HO-CDI compared to CO-CDI, had longer LOS, higher illness-severity and more healthcare exposures/surgeries, infections and antibiotic use. Tailored antimicrobial stewardship and 2-step C. difficile testing would be helpful for mitigation of both.

**Disclosures:**

All Authors: No reported disclosures

